# Oxidative Stress in Intestinal Ischemia-Reperfusion

**DOI:** 10.3389/fmed.2021.750731

**Published:** 2022-01-14

**Authors:** Guangyao Li, Shuang Wang, Zhe Fan

**Affiliations:** ^1^Department of General Surgery, The Third People's Hospital of Dalian, Dalian Medical University, Dalian, China; ^2^Department of Central Laboratory, The Third People's Hospital of Dalian, Dalian Medical University, Dalian, China; ^3^Department of Endocrinology, The Second Affiliated Hospital of Dalian Medical University, Dalian, China

**Keywords:** oxidative stress, intestinal, ischemia-reperfusion, signal pathway, review

## Abstract

Ischemia-reperfusion (I/R) injury is a manifestation of tissue or organ damage that is followed by ischemia and exacerbated by the return of blood flow to a previously damaged tissue or organ. The intestines are one of the most sensitive tissues and organs to I/R injury. Moreover, the adverse consequences of intestinal I/R (II/R) injury are not limited to the intestine itself and can also lead to damage of the distant tissues and organs. The mechanism of II/R is extremely complex and oxidative stress is the key link in the pathogenesis of II/R injury. This study summarizes the roles of oxidative stress and its signaling pathways involved in II/R. The signaling pathways that mitigate II/R injury include the nuclear factor erythroid-related factor 2 (Nrf2)-mediated signaling pathway, Wnt/β-catenin pathway, and phosphatidylinositol kinase 3 (PI3K)/Akt pathway; those that aggravate II/R injury include the Janus kinase/signal transducer and activator of transcription (JAK/STAT) pathway, Toll-like receptor (TLR) receptor-mediated signaling pathway, protein kinase CβII (PKCβII)/p66shc pathway, and microRNA (miRNA)/p66shc pathway; the effect of miRNA on related pathways and mitochondrial DNA translocation. The aforementioned pathways provide new ideas for further exploring the occurrence and development of II/R and more effective treatments for II/R injury.

## Introduction

Ischemia-reperfusion (I/R) injury is a serious clinical event. In the experimental studies and clinical practice, the ischemia itself does not cause great harm to the organism; instead, the excess reactive oxygen species (ROS) destroy cells as blood flow is restored and this shock directly causes damage to the organism (1). Intestinal I/R (II/R) injury is a common and very serious pathophysiological phenomenon (2) often caused by severe trauma, burns, shock, intestinal torsion, mesenteric thromboembolism, and small bowel transplantation (3). II/R damage is a life-threatening pathological event, which is not confined to the intestine and can cause systemic inflammatory response syndrome (SIRS) and multiple organ dysfunction syndrome (MODS), which are closely associated with the high incidence and mortality rates of various diseases (4). The mechanisms involved in II/R injury are quite complex and the response of the intestinal mucosa to I/R can be divided into the following two phases: first, tissue hypoxia and organ damage occur during ischemia; second, large amounts of ROS generated during blood flow reperfusion and reoxygenation trigger an oxidative stress response, which subsequently leads to the destruction of the intestinal mucosal barrier, increased vascular permeability, bacterial translocation, and the release of inflammatory mediators and apoptotic factors (5).

In the recent years, with more in-depth research, increasing evidence shows that oxidative stress plays a crucial role in the pathogenesis of II/R. This study focuses on the oxidative stress and II/R and a search of the Medical Subject Headings (MeSHs) was carried out using PubMed as follows: oxidative stress, intestinal, gut, and I/R. As oxygen is restored during reperfusion, abundant ROS in damaged cells and tissues can attack almost all the intracellular biomolecules (e.g., cell membranes, organelles, and even DNA) and this oxidative stress disrupts the dynamic homeostasis of the epithelial cells through signal transduction pathways, thereby resulting in the release of large amounts of inflammatory mediators and the induction of apoptosis and exacerbating the damage after reperfusion (6). Mitochondrial DNA (mtDNA) is involved in the oxidative phosphorylation of cells and maintains normal mitochondrial function. After mtDNA is disrupted, ROS production increases and the release of mtDNA into the cytoplasm induces the activation of proinflammatory mediators and proapoptotic factors (7). As the mechanisms of II/R injury have been more extensively investigated, antioxidant pathways and protection of mitochondria against damage have emerged as important means to prevent and treat II/R injury.

## Oxidative Stress

Oxygen (O_2_) molecules are indispensable for the survival of the body and they provide ATP to the body through the respiratory complex of mitochondria (8). Normal cell metabolism produces ROS, which in small or moderate amounts are beneficial for some physiological processes; however, excessive ROS production during II/R is a significant condition that leads to tissue oxidation and damage to the intestinal epithelial cells (9). ROS originate mainly from the gastrointestinal tract and although intestinal epithelial cells are protected by a mucosal barrier, pathogens can produce inflammatory factors by activating epithelial cells, polymorphonuclear neutrophils, and macrophages and these inflammatory cells release ROS and free radicals in defense against invading pathogens (10). ROS and free radicals are produced to destroy the invading pathogens, but the production of large amounts of ROS adversely affects the homeostasis of the organism and leads to oxidative damage to tissues (11).

Reactive oxygen species include both the free radical compounds and non-radical compounds such as ${\text{O}}_{2}^{-}$, OH^−^, H_2_O_2_, O_3_, ^1^O_2_, and lipid hydrogen peroxide (12). NO, NO_2_, N_2_O_3_, and ONOO^−^ are collectively referred to as reactive nitrogen species, which are usually closely associated with ROS (13). These radicals contain unpaired electrons and are, thus, highly chemically reactive toward intracellular proteins, lipids, and even DNA. The activation of ROS can irreversibly damage and deactivate target molecules (14).

Reactive oxygen species can be produced by the mitochondria, endoplasmic reticulum, cell membrane, cytoplasm, nuclei, and peroxidases and can even be produced through extracellular stress (10). Most ROS are produced by mammalian mitochondria; under normal conditions, oxygen molecules are reduced by mitochondrial cytochrome c, thereby forming water and only several oxygen molecules form ROS. Under normal conditions in the body, these free radicals are neutralized by endogenous antioxidant enzymes and, therefore, do not have adverse effects on the organism (15). The mitochondrial respiratory chain regulates ROS production (16). Mitochondrial complexes I and III generate ROS through electron leakage during oxidation (17). Mitochondrial NADPH oxidase and xanthine oxidase catalyze the conversion of O_2_ to ${\text{O}}_{2}^{-}$, myeloperoxidase (MPO) catalyzes the conversion of O_2_ to OH^−^, and mitochondrial protein kinase C (PKC) catalyzes the conversion of O_2_ to H_2_O_2_ (10). Enzymes that catalyze ROS-generating chemistry *in vivo* also include lipoxygenases, glucose oxidases, nitric oxide synthases, and cyclooxygenases (18).

Intestinal ischemia can lead to a hypoxic state involving a change in the irreversible conversion of xanthine dehydrogenase (XD) to xanthine oxidase (XO), during which reactive oxygen forms (19). After reperfusion begins and the oxygen supply is restored, electrons from XO are transferred to molecular oxygen, thus forming significant amounts of oxygen-free radicals such as O_2_·, OH, and hydrogen peroxide (H_2_O_2_), which injure DNA, cell membranes, and organelles (20). Therefore, II/R injury can decrease villus height, increase cellular infiltration, and aggravate mucosal sloughing in terms of histology. In addition, proinflammatory cytokines are released into the serum including tumor necrosis factor-α (TNF-α), interleukin 6 (IL-6), interleukin 1β (IL-1β) (21).

Although many ROS are produced during oxidative stress, the antioxidants of the body can protect cells and tissues against ROS attack to some extent. Enzymatic antioxidants [Superoxide dismutase (SOD), Glutathione peroxidase (GPX), Glutathione reductase (GSR), Catalase (CAT), and Heme Oxygenase (HO)] and non-enzymatic antioxidants (Glutathione (GSH), Thioredoxin (TRX), and melatonin) play important roles in oxidative stress (22). Exogenous substances, such as vitamin C, vitamin E, carotenoids, mineral ions (Mn and Cu), and polyphenols, also act as antioxidants (23, 24). SOD and CAT play major roles in the antioxidant defense system (25). Therefore, antioxidants scavenge excessive ROS and free radicals during the peroxidation reactions of the body and, thus, may be used to treat II/R damage.

## Signaling Pathways Involved in Oxidative Stress in II/R

### Pathways to Mitigate Oxidative Stress

#### Nuclear Factor Erythroid-Related Factor 2-Mediated Signaling Pathway

Nuclear factor erythroid-related factor 2 is a key transcription factor that regulates cellular antioxidative stress and has been found to have roles in the development of many diseases. Under normal conditions, Nrf2 binds the specific repressor protein Keap1 and forms the Nrf2/Keap1 complex, which is localized in the cytoplasm (26). When the body reacts to oxidative stress, Nrf2 dissociates from the Nrf2/Keap1 complex and enters the nucleus, where it maintains the balance of oxidation/antioxidation in the organism by upregulating the expression of antioxidants (HO-1 and SOD) (27). SOD, a specific scavenger of superoxide, catalyzes the decomposition of superoxide anions to O^2−^ and H_2_O_2_, which, in turn, is disproportionated to H_2_O and O_2_ through the action of catalase (10). Free heme is extremely lipophilic and inserts into the cell membrane of surrounding cells, consequently activating vascular endothelial cells and leading to an increase in adhesion molecules and inflammatory mediators (28). HO-1 is the enzyme responsible for heme catabolism, catalyzing the degradation of heme and the production of carbon monoxide (CO), bilirubin, and iron (29). Studies have shown that bilirubin has antioxidant effects and is an endogenous antioxidant (30). Although CO is not an antioxidant, it effectively inhibits the release of inflammatory mediators, increases the ratio of the antiapoptotic factor Bcl-2 to the proapoptotic factor Bax, and increases the survival rate in experimental animals (31). HO-1 plays an important role in II/R injury through its antioxidant effects. Because of its antioxidant effect, Nrf2 and its target genes are considered “guardians” of body tissues (32), particularly in intestinal diseases, in which Nrf2 expression improves the intestinal mucosal barrier and decreases the intestinal inflammatory response (33).

Han et al. have found that endogenous lipoxygenin A4 (LXA4) exerts antioxidant effects on II/R-induced oxidative stress by enhancing the Nrf2 signaling pathway and attenuating intestinal mucosal cell damage after I/R (34). Roberta et al. have demonstrated that cashew fruit decreases MPO activity, lipid peroxidation, and ROS by modulating Nrf2/HO-1 signaling, thus restoring antioxidant enzyme activity and significantly decreasing mortality in II/R rats (35). In addition, the use of the anti-inflammatory factor interleukin-1 receptor antagonist (IL-1Ra) not only effectively inhibits the expression of II/R inflammatory factors (IL-1β, IL-6, and TNF-α), but also markedly promotes the expression of Nrf2, HO-1, and SOD, thus decreasing intestinal tissue damage (36). Strengthening the Nrf2 pathway and promoting the nuclear translocation of Nrf2 might provide a new therapeutic pathway for treating II/R injury.

#### Wnt/β-Catenin/GSK-3β Signaling Pathway

Two Wnt signaling pathways are involved in pathophysiological process of the body. The Wnt/β-catenin signaling pathway is the classical pathway and the non-classical pathway includes the Wnt/PCP and Wnt/Ca^2+^ pathways; the most widely studied is the Wnt/β-catenin signaling pathway (37). The Wnt/β-catenin signaling pathway regulates the transcription of many target genes including inflammatory mediators, proapoptotic factors, and ROS associated with II/R injury (38). GSK-3β participates in cell proliferation, apoptosis, and the cell cycle (39). GSK-3β inhibits the Wnt/β-catenin signaling pathway, and GSK-3β phosphorylates β-catenin and forms a complex with it in the cytoplasm (40). Wnt signals inactivate GSK-3β and decrease the phosphorylation of β-catenin. The β-catenin then enters the nucleus, binds TCF and LEF, and promotes transcription (41).

Previous studies have shown that β-catenin in the cytoplasm is diminished, thereby decreasing the transcription of other genes under hypoxia (42). Study by Shin et al. has demonstrated that ROS-induced oxidative stress inhibits β-catenin transcriptional activity (43). When GSK-3β is inhibited by protein kinase B phosphorylation, mitochondrial permeability and mitochondrial membrane potential are decreased, thus attenuating ROS production and protecting mitochondria against damage (39). Zu et al. have shown that the ginsenoside Rg1 activates the Wnt/β-catenin signaling pathway and decreases II/R-induced inflammatory factors, apoptosis, and ROS (38). Activation of the Wnt/β-catenin signaling pathway may become an effective approach to attenuate II/R injury.

#### Phosphatidylinositol Kinase 3/Akt Signaling Pathway

Phosphatidylinositol kinase 3 regulates cell proliferation, differentiation, apoptosis, and stress (44). Protein kinase B (Akt), protein kinase C (PKC), and nuclear factor kappa B (NF-κB) recognize PI3K and target the activity of the downstream enzymes. When stimulated by extracellular signals, Akt is activated by transmembrane transport and phosphorylation, thereby initiating a series of intracellular reactions that regulate cellular activities (45). The threonine phosphorylation site (Thr308) and serine phosphorylation site (Ser473) on Akt protein are important factors in the activation of Akt (46). Experiments have revealed that activation of the PI3K/Akt signaling pathway protects cells against damage and decreases apoptosis during oxidative stress (47). Inhibition of Akt activation makes cells more sensitive to oxidative stress and more susceptible to damage (48). The PI3K/Akt signaling pathway plays an important role in several antioxidant responses and inflammatory cascade responses (49). Therefore, the PI3K/Akt signaling pathway is an important pathway allowing the body to maintain oxidative homeostasis.

**Figure 1:**
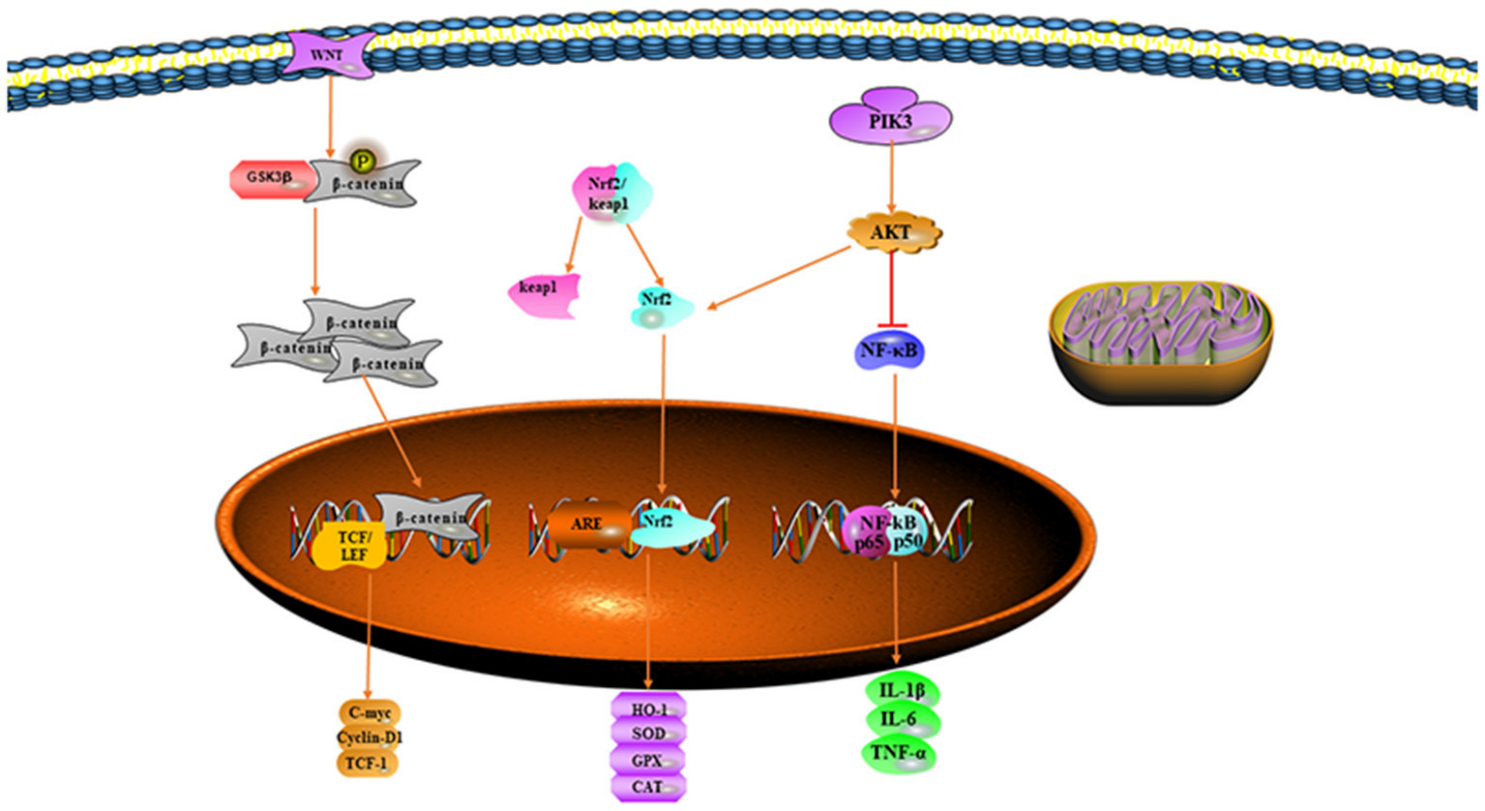
Signaling pathways mitigating oxidative stress.

Nuclear factor erythroid-related factor 2 is an important downstream target of the PI3K/Akt pathway (48). The PI3K/Akt pathway acts on Nrf2 and results in the initiation of the transcription of downstream antioxidant enzymes that protect cells against oxidative stress and inflammatory responses (50). The activation of PI3K/AKT also inhibits the inflammatory response mediated by NF-κB (51). Chen et al. have found that the ginsenoside Rg1 attenuates II/R injury by activating the PI3K/Akt pathway, thus inhibiting inflammatory responses and oxidative stress in II/R injury experiments (52). Activation of the PI3K/Akt-mediated antioxidant anti-inflammatory pathway may be a potential therapeutic option to prevent II/R injury (Figure 1).

### Pathways That Exacerbate Oxidative Stress

#### Janus Kinase/Signal Transducer and Activator of Transcription Signaling Pathway

The JAK/STAT signaling pathway is a common pathway in the signaling of many cytokines associated with cell proliferation, differentiation, apoptosis, and inflammation (53). JAKs are a class of non-receptor-type tyrosine kinases. After a cytokine binds its cognate receptor, STATs are activated by phosphorylation of JAKs and the resulting dimer subsequently enters the nucleus and regulates the transcription of target genes (54). Although the JAK/STAT pathway is known to be associated with I/R injury, its effects on I/R injury are quite different. Zhang et al. have suggested that activation of the JAK/STAT signaling pathway by using dexmedetomidine inhibits II/R injury by decreasing caspase-3 and Bcl-2/Bax ratios, thus significantly decreasing apoptosis (53). However, other studies have found that activation of JAK/STAT signaling leads to severe damage to the mucosa after intestinal I/R injury (55). STAT signaling acts on intracellular ROS via the JAK2 pathway and activation of JAK/STAT by oxidative stress may exacerbate II/R injury; in contrast, inhibitors of the JAK/STAT signaling pathway, such as pyruvate, inhibit oxidative stress, decrease neutrophil granulocyte infiltration, regulate microcirculation, and inhibit apoptosis (56). The role of the JAK/STAT signaling pathway in II/R injury requires further research to be clarified.

#### Toll-Like Receptor-Mediated Signaling Pathways

Toll-like receptors are a family of receptors expressed in cell membranes and associated with the recognition of pathogenic microorganisms by the immune system, thus allowing for the transmission of extracellular antigen recognition information to the cell (57). As pattern recognition receptors, TLRs recognize not only pathogen-associated molecular patterns (also known as exogenous ligands of TLRs), such as viruses, bacteria, fungi, and other pathogenic microorganisms (57), but also damage-associated molecular patterns (also known as exogenous ligands for TLRs), which are actively secreted or passively released by the organism during stress, as well as molecular patterns (also known as endogenous ligands for TLRs), such as low-density lipoproteins and heat shock proteins, apoptotic cells, and nucleic acids (58). During II/R, excessive ROS also stimulates the activation of TLRs and consequently aggravates the inflammatory response (59). Activated TLRs trigger a systemic inflammatory response by binding to the C-terminus of the junction protein MyD88 and then to downstream target genes [mitogen-activated protein kinase (MAPK) and NF-κB] through the N-terminal end of the MyD88 molecule, thus inducing the release of inflammatory mediators (IL-1β, IL-6, and TNF-α) (60). In most models of intestinal inflammation, TLR4 induces inflammatory responses more commonly than other TLRs. Marwan has shown that cinnamaldehyde pretreatment attenuates oxidative stress by restoring the levels of SOD, GSH, Lactic Dehydrogenase (LDH), and Malondialdehyde (MDA) in I/R-treated intestinal tissues; it also attenuates intestinal injury by inhibiting the expression of p65 and p50 in the NF-κB pathway (61). TLR-mediated inflammatory responses and apoptosis clearly have strong adverse effects on cells and tissues in II/R; therefore, targeting TLR pathways for inhibition may be a good choice for treating II/R injury.

#### Protein Kinase CβII/P66shc Pathway

Protein kinase C is a multifunctional protein kinase that is normally present in the cytoplasm. When cells are stimulated, PKC translocates from the cytoplasm to the cell membrane in the form of Ca^2+^, where it plays an important role in signal transduction pathways through phosphorylation (62). Among the various PKC isoforms, PKCβII is activated in II/R injury (63). The bridging protein p66shc is an important regulator of oxidative stress in mitochondria and cells. When oxidative stress occurs, p66shc is phosphorylated and after dephosphorylation by protein serine-threonine phosphatase, it translocates to mitochondria, where it promotes ROS production (64). Phosphorylated p66shc promotes the phosphorylation of FOXO3a, which becomes inactive when phosphorylated, and active FOXO3a upregulates the expression of the antioxidant enzyme SOD (65). In addition, p66shc causes the release of mitochondrial cytochrome c. Cytochrome c entering the cytoplasm leads to the activation of the proapoptotic factors caspase-3, 6, and 9 (66, 67). Wang has shown that LY333531, which inhibits the PKCβII/p66shc pathway, attenuates damage to the intestines in II/R (68). Therefore, inhibition of the PKCβII/p66shc pathway, which not only decreases ROS production but also inhibits the activation of proapoptotic factors, has a significant effect on the treatment of II/R injury.

#### Effect of microRNA on Mechanisms Involved in II/R Injury

MicroRNA is a type of highly conserved single-stranded non-coding RNA widely found in plants and animals. It negatively regulates the expression of target genes after transcription through complementary pairing with mRNA (69). In the recent years, numerous studies have found that II/R increases or decreases miRNA expression levels in intestinal tissues and these effects play varying roles in II/R damage depending on the mechanism of action of downstream target genes (70). Numerous studies have shown that miR-23a-5p (71), miR-34a-5p (66), and miR-351-5p (72), isoforms of miRNAs, affect II/R injury to some extent by regulating a series of signal transduction pathways.

Sirtuin1 (SIRT1) is a histone deacetylase whose function depends on nicotinamide adenine dinucleotide. Because of its important targets, SIRT1 has roles in regulating cellular metabolism, senescence, redox homeostasis, and apoptosis (73). SIRT1 mediates oxidative responses by regulating transcription factors of antioxidant genes. It binds the promoter of p66Shc, deacetylates p66Shc, and decreases the transcription and translation of p66shc, thereby regulating ROS levels and inducing apoptosis (74). Owing to the antioxidant effect of SIRT1, numerous studies have linked SIRT1 to I/R injury, implying that SIRT1 plays a key role in II/R-induced oxidative stress (75). Wang et al. have experimentally found that miR-34a-5p inhibits SIRT1-mediated signaling pathways, thus leading to the upregulation of p66shc, the reduction of MnSOD and the activation of caspase-3, and exacerbating II/R injury (66).

**Figure 2:**
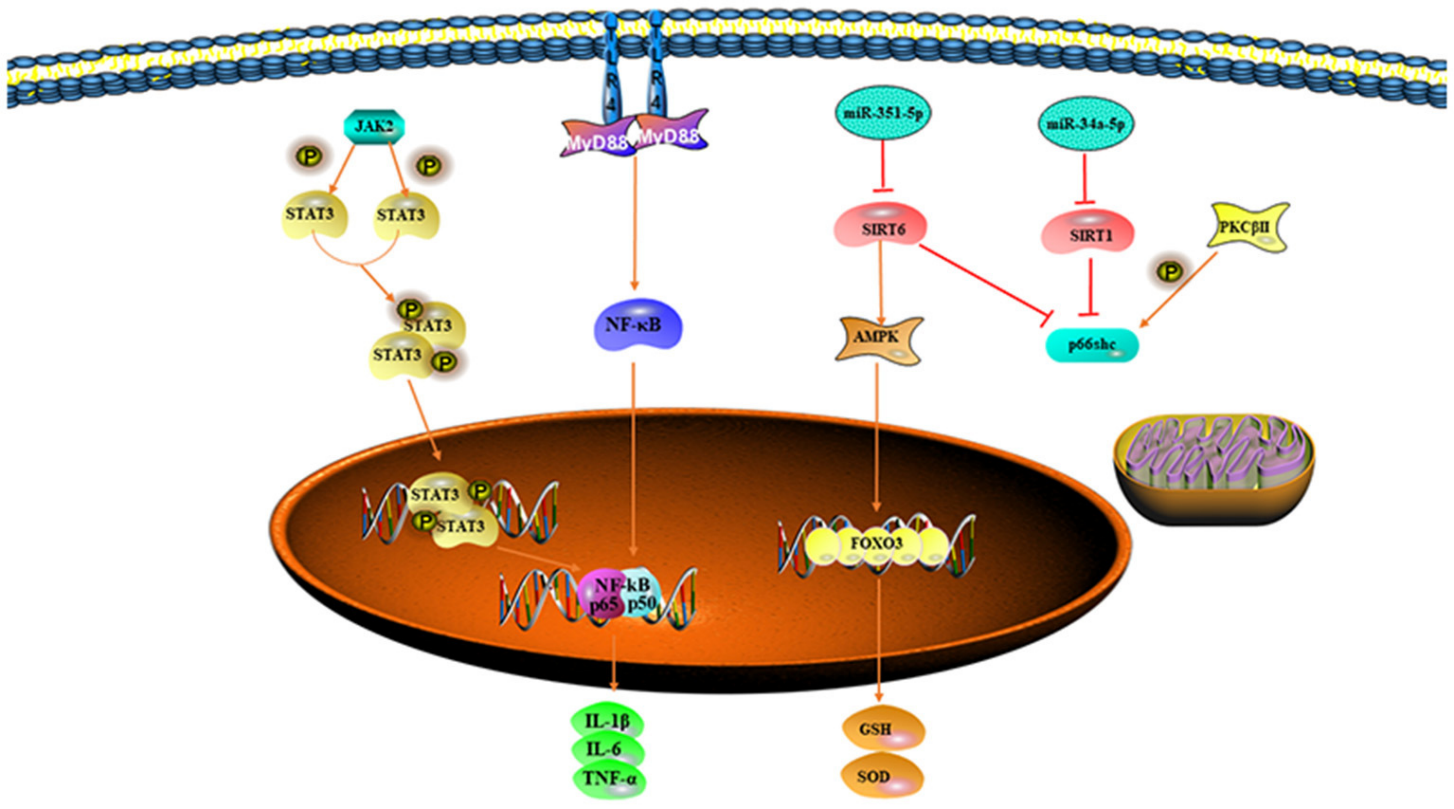
Signal pathways that exacerbate oxidative stress.

Mitogen-activated protein kinase 13 is a type of p38 MAPK that links extracellular signals to intracellular signals and regulates processes such as apoptosis, the inflammatory response, differentiation, and senescence (76). SIRT6 is a member of the sirtuin family that is involved in the cellular processes such as inflammatory responses, oxidative stress, and apoptosis (77). SIRT6 decreases apoptosis by binding specific sites in the Bax promoter and it improves the antioxidant capacity of the body by activating FOXO3a, either directly or through activation of Adenosine 5′-monophosphate (AMP)-activated protein kinase (AMPK) (78). Hu et al. have experimentally found that MAPK13 and SIRT6 are the downstream target genes of miR-351-5p. During II/R, miRNA-351-3p is significantly upregulated and MAPK13 and SIRT6 expression are significantly decreased, thus leading to the activation of their downstream inflammatory response, oxidative stress, and apoptotic signaling pathways and increasing the degree of intestinal damage (72) (Figure 2).

## Mitochondrial DNA Translocation Aggravates Oxidative Stress

Mitochondria, important organelles in eukaryotic cells, are involved in energy supply, apoptosis, maintenance of intracellular ion homeostasis, and the production and removal of ROS. When pathogenic microorganisms infect the host, mitochondria have important roles in organisms by inducing multiple immune responses to clear the infected cells (79). mtDNA, genetic material located within the inner membrane of mitochondria, is involved in oxidative phosphorylation as well as maintaining normal mitochondrial function (80). Oxidative stress is likely to damage mtDNA because of proximity of the mtDNA to the respiratory chain and its lack of protective histones (81). After oxidative stress damage occurs, the continual increase in damaged mtDNA destroys the function of mitochondria. mtDNA copy and gene transcription levels decrease, thereby affecting the formation of the respiratory chain complex and leading to mitochondrial dysfunction. Mitochondrial dysfunction, in turn, promotes massive production of ROS, in a cycle that eventually leads to apoptosis (82).

**Figure 3:**
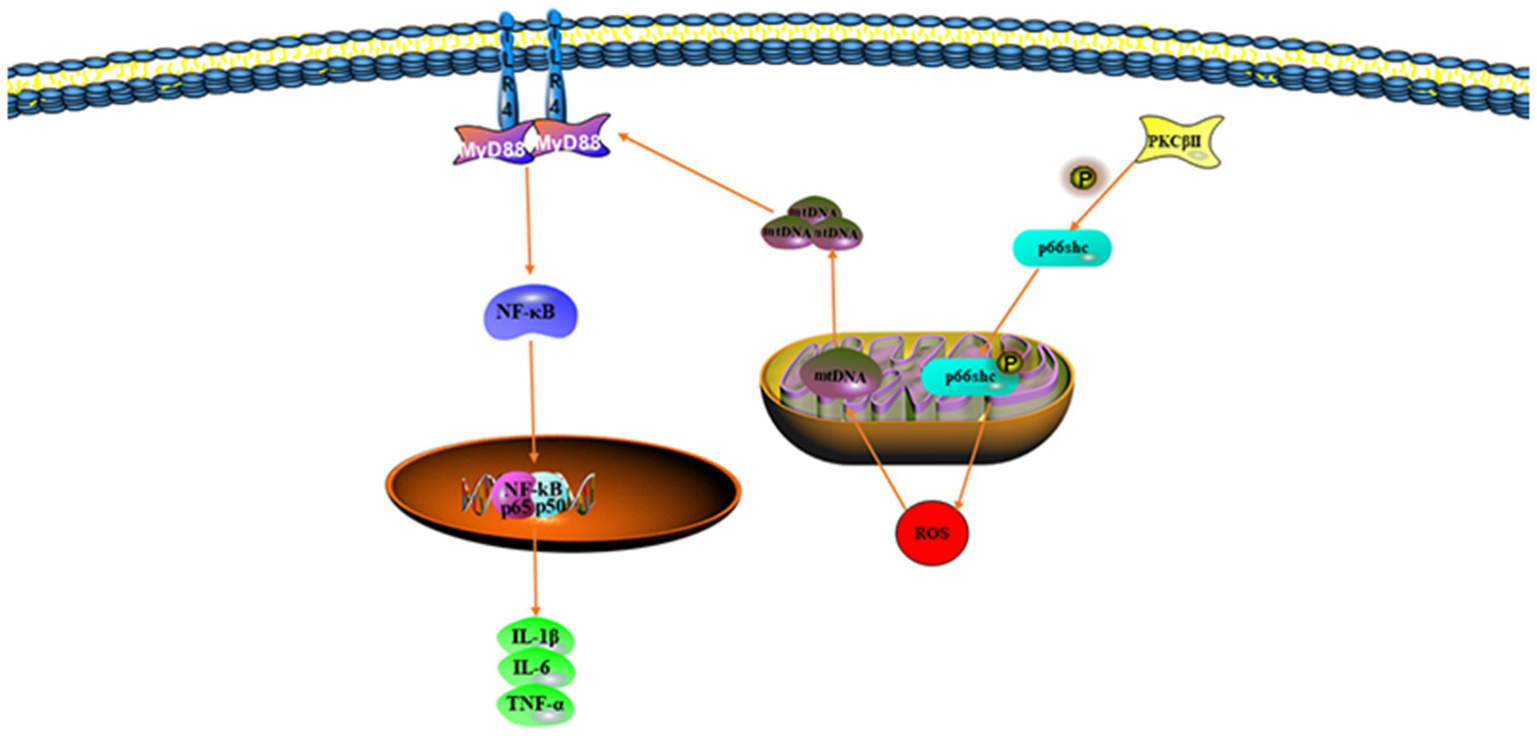
Mitochondrial DNA (mtDNA) translocation aggravates oxidative stress.

During II/R injury, the body initiates damage-associated molecular patterns, many of which are derived from mitochondria (83). Normally, mtDNA is present between the inner and outer mitochondrial membranes. However, during II/R, mitochondria are stimulated by oxidative stress and mtDNA fragments are released into the cytoplasm, thereby triggering a series of inflammatory responses (84). Several studies have shown that mtDNA damage results in the release of inflammatory factors (IL-1β, IL-6, and TNF-α) through TLRs and the NOD-like receptor protein 3 (NLRP3) signaling pathway (85). Hu et al. have found that the mitochondria-targeted antioxidant MitoQ decreases mitochondrial ROS production through activation of the Nrf2 pathway, decreases oxidative stress, and improves mtDNA translocation caused by II/R damage (86) (Figure 3).

## Conclusion

Intestinal ischemia-reperfusion injury is a pathological condition involving multiple organs; reperfusion is necessary to maintain homeostasis, but reoxygenation during reperfusion produces great damage to the organism. Excessive oxidative stress can lead to damage to cell membranes, organelles, and even DNA. Therefore, decreasing ROS production and strengthening the antioxidant defense system play important roles in the treatment of II/R injury. However, the mechanism of ROS production and the molecular mechanism of ROS that leads to tissue damage is very complex (Figure 1). With the continual progress of related research, a breakthrough has been achieved in treating excessive ROS and decreasing oxidative stress. Even greater breakthroughs are soon expected in the development, prevention, and treatment of II/R injury.

## Author Contributions

GL, SW, and ZF contributed to conception and design of the article. GL wrote the first draft of the manuscript. SW and ZF wrote sections of the manuscript and revised the manuscript. All authors contributed to manuscript revision, read, and approved the submitted version.

## Funding

Information retrieval was done by the National Natural Science Foundation of China (81701965) and the Natural Science Foundation of Liaoning Province (2020-BS-187).

## Conflict of Interest

The authors declare that the research was conducted in the absence of any commercial or financial relationships that could be construed as a potential conflict of interest.

## Publisher's Note

All claims expressed in this article are solely those of the authors and do not necessarily represent those of their affiliated organizations, or those of the publisher, the editors and the reviewers. Any product that may be evaluated in this article, or claim that may be made by its manufacturer, is not guaranteed or endorsed by the publisher.
